# The Swedish COG6‐CDG experience and a comprehensive literature review

**DOI:** 10.1002/jmd2.12338

**Published:** 2022-09-21

**Authors:** Zhi‐Jie Xia, Bobby G. Ng, Elizabeth Jennions, Maria Blomqvist, Anneli Sandqvist Wiklund, Carola Hedberg‐Oldfors, Carlos Rodriguez Gonzalez, Hudson H. Freeze, Sofia Ygberg, Erik A. Eklund

**Affiliations:** ^1^ Sanford Burnham Prebys Medical Discovery Institute La Jolla California USA; ^2^ Department of Pediatrics Institute of Clinical Sciences, Sahlgrenska Academy Gothenburg Sweden; ^3^ Department of Laboratory Medicine Institute of Biomedicine, University of Gothenburg Gothenburg Sweden; ^4^ Department of Clinical Chemistry Sahlgrenska University Hospital Gothenburg Sweden; ^5^ Department of Psychology Karolinska University Hospital Stockholm Sweden; ^6^ Department of Medical Biochemistry and Biophysics Karolinska Institute Stockholm Sweden; ^7^ Centre for Inherited Metabolic Diseases (CMMS) Karolinska University Hospital Stockholm Sweden; ^8^ Pediatric Neurology Karolinska University Hospital Stockholm Sweden; ^9^ Pediatrics, Clinical Sciences Lund University Lund Sweden; ^10^ Pediatric Neurology Skåne University Hospital Lund Sweden

**Keywords:** Brefeldin A, COG6, congenital disorders of, enamel hypoplasia, glycosylation, glycosylation, hypohidrosis

## Abstract

Here, we present the first two Swedish cases of Conserved Oligomeric Golgi complex subunit 6‐congenital disorders of glycosylation (COG6‐CDG). Their clinical symptoms include intellectual disability, Attention Deficit/Hyperactivity Disorder (ADHD), delayed brain myelinization, progressive microcephaly, joint laxity, hyperkeratosis, frequent infections, and enamel hypoplasia. In one family, compound heterozygous variants in *COG6* were identified, where one (c.785A>G; p.Tyr262Cys) has previously been described in patients of Moroccan descent, whereas the other (c.238G>A; p.Glu80Lys) is undescribed. On the other hand, a previously undescribed homozygous duplication (c.1793_1795dup) was deemed the cause of the disease. To confirm the pathogenicity of the variants, we treated patient and control fibroblasts with the ER‐Golgi transport inhibitor Brefeldin‐A and show that patient cells manifest a significantly slower anterograde and retrograde ER‐Golgi transport.

**Synopsis**

We describe two novel conserved oligomeric Golgi‐congenital disorders of glycosylation (COG6‐CDG) individuals, extensively review the current literature and show that Brefeldin‐A treatment of patient fibroblasts can be used to prove pathogenicity of genetic variants of uncertain significance in the *COG6* gene.

## 1 INTRODUCTION

Congenital disorders of glycosylation (CDG) comprise some 160 different genetic conditions^1^ with the common feature of dys‐ or hypoglycosylation of proteins and/or lipids.^2^, ^3^ These syndromes are diverse in clinical expression, ranging from mild phenotypes to severe syndromes with early death. Common findings include failure to thrive, hypoglycemia, muscular hypotonia, developmental delay (DD)/intellectual disability (ID), epilepsy, cerebellar atrophy/hypoplasia with ataxia, liver disease, coagulopathy, retinopathy, and multiple endocrinopathies.^4^ An important subgroup is the Conserved Oligomeric Golgi (COG)‐CDG syndromes, where the genetic defect disrupts the function of the COG complex, involved in maintaining the homeostasis of the Golgi by regulating retrograde Golgi transport.^5^ The COG complex contains eight subunits, of which deficiencies in seven (all but COG3) have been described to cause a CDG.^5^ There are 16 publications totaling 41 patients with COG6‐CDG, of which 30 have clinical descriptions and 16 derive from two families (12 + 4).^6^, ^7^, ^8^, ^9^, ^10^, ^11^, ^12^, ^13^, ^14^, ^15^, ^16^, ^17^, ^18^, ^19^, ^20^, ^21^ Of the 41, only 9 presented with compound heterozygous variants, whereas the others were homozygous. The phenotype of COG6‐CDG varies between subjects, but is severe in a significant proportion with growth retardation, microcephaly, DD or ID, hypohidrosis, arthrogryposis, and 17/41 fatalities during the first 15 months of life. In this report, we describe the first two Swedish patients, including the first COG6‐CDG patient of North European and South American descent, present a novel mutation, and biochemical data strengthening the genetic findings.

## 2 METHODS

### 2.1 Whole‐genome sequencing

Clinical WGS for Patient 1 (P1) was performed at the Karolinska University Laboratory, Stockholm and interpreted using the pipeline recently described.^22^ The WGS analysis of Patient 2 (P2) was performed at Clinical Genomics, SciLife, Stockholm and analyzed using the in silico panel from the Centre for Inherited Metabolic Diseases (CMMS), Karolinska University Hospital (dbCMMSv13).

### 2.2 LC–MS analysis of transferrin

The LC–MS analysis of transferrin was performed at the Clinical Department in Lund, Sweden, as described previously.^23^ Briefly, columns with polyclonal anti‐transferrin antibodies conjugated to POROS‐aldehyde self‐pack medium were used to purify transferrin from sera. The eluates were concentrated on an analytical C4 monolith column and then analyzed on a Thermo Scientific Q Exactive quadrupole‐orbitrap mass analyzer. The mass spectrometer was operated in full scan mode and data was deconvoluted using Pro Mass 2.8.2 (Novatia, LLC, Newton, PA, USA).

### 2.3 Brefeldin A‐induced retrograde and anterograde assay

Fibroblasts from two controls and the two subjects were subjected to brefeldin A (BFA)‐induced retrograde transport assay as previously described with slight modifications.^24^ Briefly, cells were incubated with prewarmed normal growth medium containing 0.25 μg/ml BFA for 0, 6, 12, 18, 24, 30, 36, and 42 min at 37°C. The incubations were stopped by washing cells with ice‐cold Dulbecco's phosphate‐buffered saline (DPBS) and cells were fixed with 4% paraformaldehyde for 10 min at room temperature. Cells were permeabilized and stained by Alexa Fluor 488 anti‐Giantin antibody (BioLegend, San Diego, CA, USA). The percentage of cells with ER staining was determined at the given time points. Anterograde transport assay was performed as previously described.^24^ Briefly, fibroblasts from two unaffected controls and two patients were grown on glass coverslips for 2 days. After incubating the cells with 0.25 μg/mL BFA for 1 h at 37°C, cells were washed with DPBS twice, then switched to new plates with prewarmed normal growth medium and incubated for 0, 20, 40, 60, 80, 100, and 120 min at 37°C. The incubations were stopped and processed as described above.

### 2.4 Protein abundance of COG subunits in lobe B

There are four COG subunits in COG complex lobe B, named COG5, COG6, COG7, and COG8. To analyze the protein abundance of all lobe B subunits, human fibroblast cells were harvested using SDS lysis buffer.^25^ The lysates (10 μg of protein) obtained from each cell line were fractionated by SDS‐PAGE on 8% gels and immunoblotted with rabbit polyclonal COG subunit antibodies (COG antibodies provided by Dr. Daniel Ungar, University of York, UK). The blots were developed using a SuperSignal West Dura enhanced chemiluminescence kit (ThermoFisher Scientific, Waltham, MA, USA) according to the manufacturer's instructions, and quantified by ImageJ (National Institutes of Health, Bethesda, MD, USA).

### 2.5 Statistical analysis

Only descriptive statistics were used in the data analysis.

## 3 RESULTS

Table 1 is a summary of demographic and clinically relevant data for P1 and P2 compared with previously reported cases. Major findings in this CDG include structural brain abnormalities, intellectual disability/developmental delay, muscular hypotonia, facial dysmorphia, malformations of the gastrointestinal, urogenital and cardiac systems, liver, and coagulation abnormalities, hypohidrosis, enamel hypoplasia, and arthrogryposis.

### 3.1 Patient 1

This patient is the second child of unrelated parents of North European and Colombian descent. She was born at term, after a normal pregnancy, with a birth weight of 3110 g (−1 SD), length 49.5 cm (0 SD), and head circumference 33 cm (−2 SD). She was referred for neuropediatric evaluation at 9 months of age, due to delayed milestones, poor growth, and hypotonia. Metabolic investigation showed normal results including ammonia, amino acids, and acylcarnitines. INR was 1.2 (normal range <1.2) and serum transaminases and gamma‐glutamyltransferase were normal. There were no signs of immunodeficiency and a normal complete blood count. Magnetic resonance imaging (MRI) of the brain at 12 months showed delayed myelination. Whole‐genome sequencing (WGS) identified two variants in *COG6* (NM_020751.2); c.785A>G (p.Tyr262Cys) from the father, and c.238G>A (p.Glu80Lys) from the mother. The first variant is previously described,^18^ and the second was novel, but predicted pathogenic. Routine analysis of carbohydrate‐deficient transferrin (CDT) by high‐pressure liquid chromatography (HPLC) showed a normal result, but a type 2 pattern was detected using mass spectrometric analysis of immunoprecipitated transferrin.^23^ Detailed examination revealed several typical symptoms, including plantar hyperkeratosis, enamel defects with dark discoloration of the teeth, hypohidrosis, and recurrent hyperthermia. A basic coagulation investigation, and ultrasound of the liver and heart were all apparently normal. Her growth continues to be poor (weight − 3 SD and height − 2 SD at 8 years of age), and she has had a progressive microcephaly (44.2 cm [−3.5 SD] at 2.5 years of age). Independent walking was established at 2.5 years of age.

A full neuropsychological and motor evaluation was performed at 4:9 years. The results showed a general developmental delay in motor, language, communication, and cognitive function, in general corresponding to half her age (20–30 months developmental age at the age of 58 months). Cognition was assessed using WPPSI‐IV and Vineland‐II. The whole scale result was 47 IK (CI 43–55; <0.1 percentile), with relative strengths in practical and social skills.

According to the Diagnostic and Statistical Manual of mental disorders, fifth edition (DSM‐V) the diagnosis moderate intellectual disability and unspecified Attention Deficit/Hyperactivity Disorder (ADHD) were established.

Currently, she continues to develop slowly, but she has not lost any function.

### 3.2 Patient 2

P2 is the third child of consanguineous Syrian parents, with a history of multiple children showing similar symptoms. The first child in the family died at 10 months of age (in Syria) of unknown cause, but had DD, microcephaly, and seizures according to the parents. P2 was born after a normal pregnancy at 33 weeks of gestation with a birth weight of 2200 g (0 SD) and a head circumference of 29 cm (−2 SD). After birth, he developed transient respiratory distress syndrome, hypoglycemia, and jaundice. He was discharged at 37 weeks of corrected age and did not attend planned follow‐ups.

He presented again at 1 ear of age, with microcephaly and DD, and was at a developmental level of 6–8 months in all domains but had not regressed. Neurological examination showed a progressive microcephaly (head circumference − 5.5 SD) and low tone with a prominent metopic suture. He demonstrated plantar and palmar hyperkeratosis, splenomegaly, chronic diarrhea, enamel hypoplasia, and failure to thrive with a height and weight under −2 SD. A full formal neuropsychological assessment has not been performed yet.

Some liver function tests were abnormal with an ALT of 1.98 μkat/l (normal range <0.9), AST 8.0 μkat/l (<0.9) and gamma‐glutamyl transpeptidase (GGT) of 2.05 μkat/l (0.11–0.27). INR was raised (1.4; normal range 0.8–1.2) and did not respond to vitamin K. He also had a persistent thrombocytopenia (lowest value 59 10^9^/L) with otherwise normal blood parameters and raised alpha‐fetoprotein of 8820 μg/L (normal range < 10). Baseline tests of immune function were normal.

Abdominal ultrasound showed a normal‐sized liver with multiple small hypoechogenic lesions and splenomegaly. A liver biopsy revealed noninflammatory cirrhosis. Brain MRI and cardiac evaluation with ECHO and ECG were normal. Metabolic investigations showed a type 2 pattern of serum transferrin by HPLC and WGS showed a homozygous 3‐bp duplication in *COG6* (NM_020751.2); c.1793_1795dup; p.(Pro598dup). Both parents are heterozygous carriers of the variant.

At the last visit at 3.5 years of age, he had continued to make developmental progress without regression. He could walk and run independently though he had frequent falls. He had a mature pincer grip bilaterally and could make a tower of two bricks. He was able to follow two‐step instructions but had an expressive language delay. He had had recurrent admissions with viral infections. Continued follow‐up of his cirrhosis has shown decreasing transaminases, stable INR, normal albumin, and a continued thrombocytopenia. His head circumference was −6 SD, and height, and weight −2 SD.

### 3.3 Variants in COG6 affect lobe B subunit protein level

**FIGURE 1:**
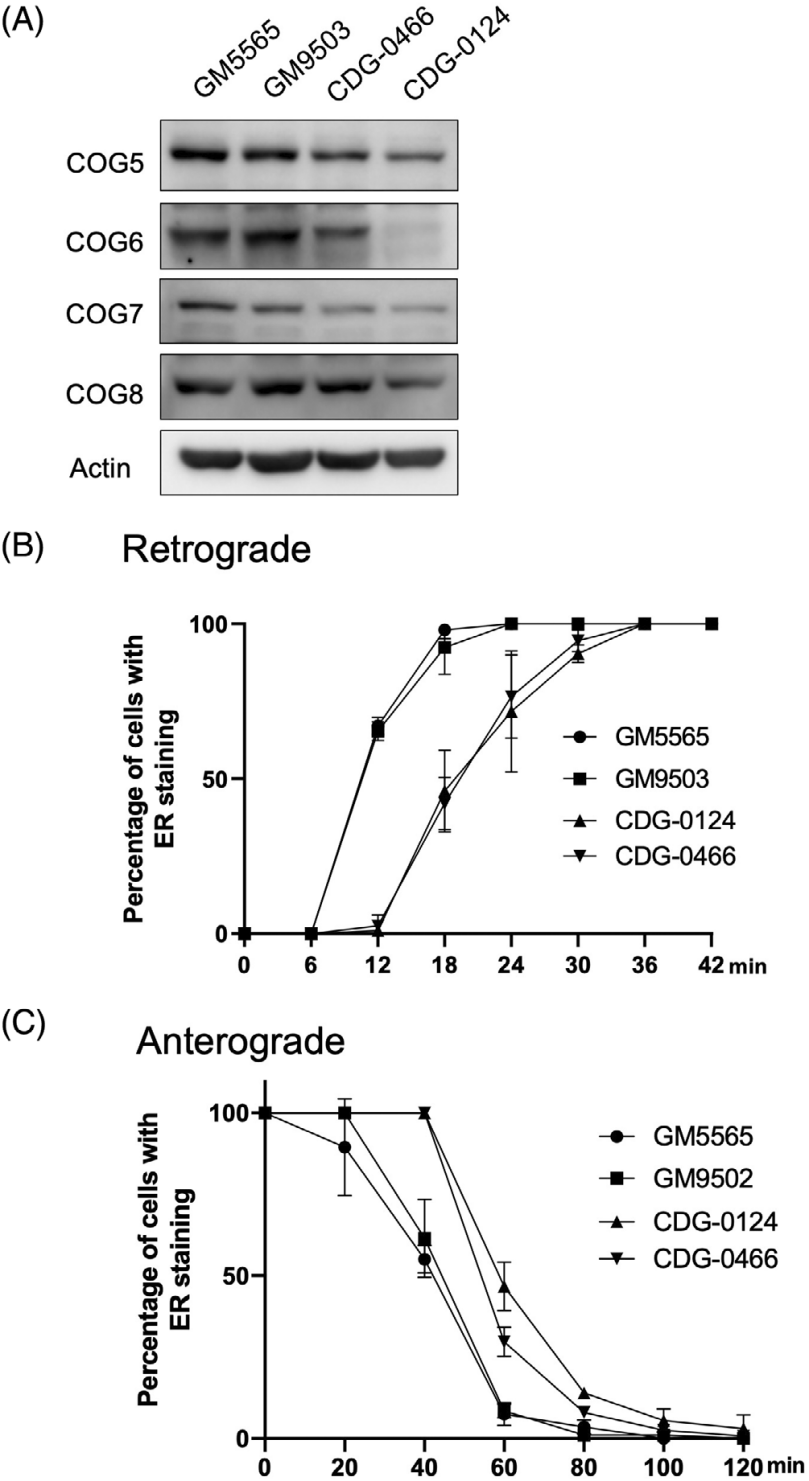
Analysis of the effects on COG complex lobe B subunit levels and antero‐ and retrograde ER‐Golgi transportation in control (GM5565 and GM9503) and patient fibroblasts. (A) Representative Western Blot images of the four COG complex subunits in lobe B. Experiments were done in triplicate. P1 is CDG‐0466 and P2 is CDG‐0124. (B) BFA‐induced retrograde transport. (C) Anterograde transport after BFA washout. Giantin was used as a Golgi marker. Eighty to 100 cells were counted in each time point. Percentage of ER staining pattern was counted for each time point. Experiments were done in triplicates

P1 had an approximately 30% depletion of COG6 protein (Figure 1A). P2 shows a more than 90% reduction of COG6 protein level (Figure 1A). The steady‐state level of other COG complex subunits in lobe B were also tested. COG7 expression was affected the most with around 50% depletion in both patients. Interestingly, we observed a drastic decrease of COG8 in P2 fibroblasts where COG6 was nearly absent. COG5 also showed some decrease, especially in P2 fibroblasts.

### 3.4 BFA‐induced retrograde and anterograde transport assay

In the retrograde transport assay (moving from the Golgi to the ER) after BFA treatment, we observed a significant delay in both patients' fibroblasts, which is a typical abnormality in COG‐CDGs (Figure 1B). When BFA is washed out, the Golgi will reform, providing a situation to study protein trafficking from ER to the Golgi. Like other published COG‐CDGs, we observed a delayed anterograde transport of the Golgi marker, Giantin from the ER to Golgi in both patients (Figure 1C).

## 4 DISCUSSION

**FIGURE 2:**
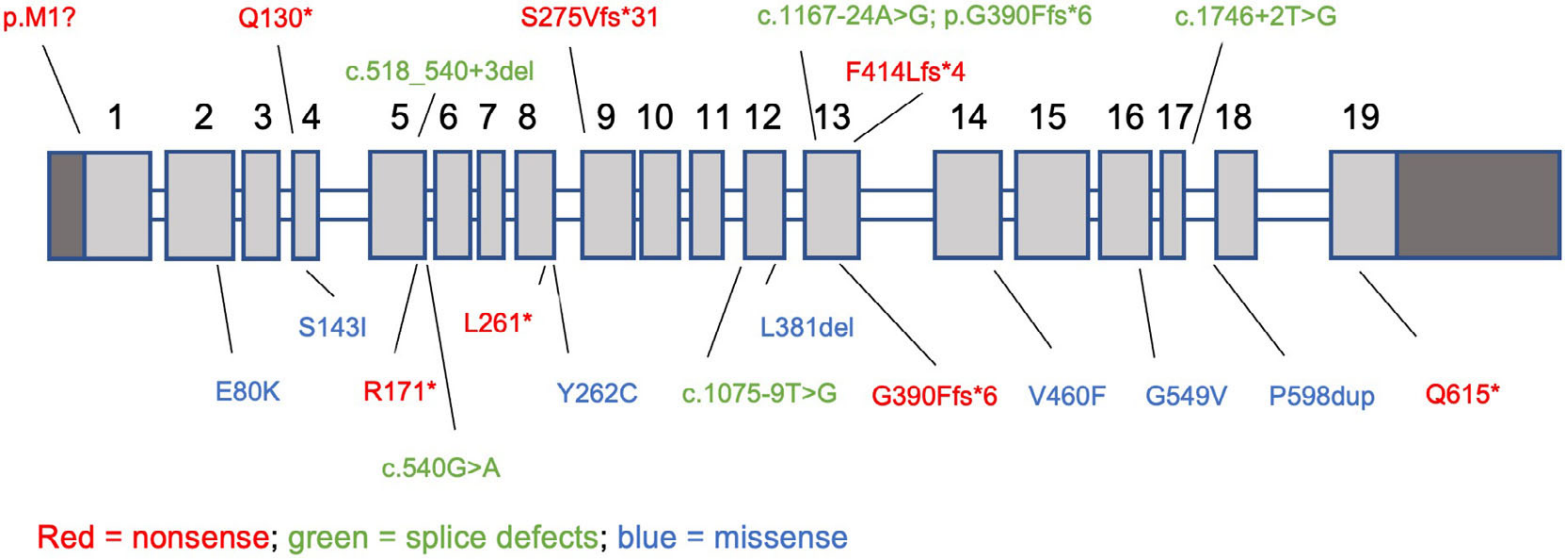
Mutational spectrum of COG6‐CDG. A schematic representation of all published mutations causing COG6‐CDG. Non‐sense mutations are in red, splice mutations in green and missense mutations in blue. (*COG6*; NM_020751.3)

In this article, we describe the clinical phenotypes and biochemical confirmation of two individuals carrying pathogenic variants in *COG6*. To compare their phenotypes to the previously published cases a thorough literature review was performed, summarized in Table I. Most known patients originate from the Middle East and the countries surrounding the Mediterranean Sea, whereas P1 is the first known patient from Northern Europe and South America. There are, including the cases presented in this report, 20 reported pathogenic variants, where the two most frequently seen, c.1167‐24A>G, causing p.Gly390Phefs*6, and c.511C>T, causing p.Arg141*, represent 14 and 10 alleles, respectively. The mutations divide in seven missense, eight nonsense, and five splice variants (Figure 2). Homozygosity for p.Arg141* causes a particularly severe phenotype where the four published cases died within 40 days of age,^7^, ^11^, ^18^ whereas Gly390Phefs*6 has a much milder clinical presentation (also known as Shaheen syndrome^19^). Homozygous variants, suggesting consanguinity, were found in 32/43 patients.

**TABLE I:** Demographic and clinical summary of all published COG6‐CDG patients including the ones reported in this article

	Case																													
	P1	P2	R1:1	R2:1	R3:1	R4:1	R5:1	R6:1	R6:2	R7:1	R7:2	R8:1	R9:1	R10:1	R11:1	R11:2														
Demography																													
Sex	F	M	F	F	F	M	M	F	F	M	M	M	F	M	M	M														
Origin	Swedish/ Columbian	Syrian	Italian	Albanian	Chinese	Moroccan	Moroccan	Greek	Greek	Israeli (Arabic)	Israeli (Arabic)	Chinese	Saudi	Chinese	Saudi	Saudi														
Deceased	Alive	Alive	14 months	40 days	Alive	12 months	9 months	4 days	15 days	18 days	30 days	Alive	Alive	Deceased, age unknown	3 months	Alive														
Genetics																													
Gene variant	238G>A/ 785A>G	hz. 1793_ 1795dup	823delA/1141_1143delCTC	hz. 511C> T	428G>T/ 1843C>T	hz.782 T>A	hz.782T>A	hz. 511C>T	hz. 511C>T	hz. 518_540 + 3del	hz. 518_540 +3del	1A>G/ 388C>T	hz. 1167‐24A>G	511C>T/ 540G>A	hz. 1378G>T	hz. 1167‐24A>G														
Protein consequence	Glu80Lys/Tyr262Cys	Pro598dup	Ser275Valfs* 31/Leu381del	Arg171*	Ser143Ile/ Gln615*	Leu261*	Leu261*	Arg171*	Arg171*	frameshift + splice defect	frameshift + splice defect	Met1?/ Gln130*	Gly390 Phefs*6	Arg171*/ Glu180Glu	Val460Phe	Gly390 Phefs*6														
gnomAD frequency	0/4E−6	0	1.19e−5/ 1.21e−5	3.2E−5	0/0	0	0	3.2E−5	3.2E−5	0	0	4.7E−6/0	0	3.2E−5/0	3.6E−5	0														
Pregnancy/perinatal																													
Pregnancy	Normal	Normal	N/R	N/R	N/R	IUGR	IUGR	Oligo/IUGR	N/R	Oligo/IUGR	Poly‐hydramnios	N/R	IUGR	N/R	Hydrops fetalis	N/R														
Gestational age at birth	38	33	37	32	39	37	38	30	Premature	38	37	40	34	34	Premature	Term														
Peri−/neonatal event	−	RDS, hypoglycemia, jaundice	N/R	Postnatal resuscitation	−	Urgent C sec, Apgar 4–7‐8	C sec, Apgar 6–7, CPAP	C sec, postnatal intubation	Postnatal intubation	C section	C section	C section	−	C section	N/R	N/R														
Growth																												
Head circumference	−4 SD	−5.5 SD	<P3	Microcephaly	<P3	<P1	<P3	P10‐P25	N/R	Microcephaly	− 2.5 SD	−2 SD	−2.2 SD	N/R	Micro‐cephaly	Microcephaly														
Length	−2 SD	−3 SD	<P3	N/R	<P3	<P1	<P1	P3‐10	N/R	N/R	N/R	− 0.4 SD	−2.5 SD	N/R	N/R	N/R														
Weight	−3 SD	−2 SD	<P3	<P1	P3–P10	<P1	<P1	P10	N/R	−4 SD	−3 SD	−2 SD	−2.1 SD	N/R	FTT	N/R														
Neurological																											
Brain MRI	Delayed myelination	Normal	Corpus callosum hypoplasia, ventricle enlargement	N/R	Reduced WM, brain atrophy, ventricle enlargement	Cerebellar hypoplasia	Dysgyration, enl ventricles, cc/vermis hypoplasia	N/R, US of brain normal	N/R	Delayed myelination, WM loss, thin corpus callosum	Intrauterine US, partial agenesis of CC	Unspecific changes	Brain atrophy, thin corpus callosum	Enlarged ventricles	Agenesis of corpus callosum, cerebellar hypoplasia	Hypomyelination, cerebellar hypoplasia														
Developmental delay (DD)/ intellectual disability (ID)	Intermediate ID	DD	Severe DD	Indeterminable	Severe ID	Severe DD	Severe DD	Indeterminable	Indeterminable	Indeterminable	Indeterminable	N/R	DD/speech delay	Indeterminable	DD	DD														
Seizures	−	−	−	−	−	N/R	GTC	−	N/R	−	−	Convulsions/normal EEG	−	N/R	−	seizures														
Muscular tone	−	Hypotonia	Hypotonia	N/R	Hypotonia	Hypotonia	Hypotonia	Hypotonia	−	Hypotonia	Hypotonia	−	Hypotonia	Hypotonia	Hypotonia	Hypotonia														
Muscular	−	−	N/R	N/R	N/R	−	−	N/R	N/R	N/R	N/R	↑ CK/CKMB	−	N/R	N/R	N/R														
Neuropsychiatric	ADHD	−	Indeterminable	Indeterminable	Indeterminable	Indeterminable	Indeterminable	Indeterminable	Indeterminable	Indeterminable	Indeterminable	N/R	N/R	Indeterminable‐able	N/R	N/R														
Ophthalmological	−	−	N/R	N/R	N/R	N/R	−	Incomplete eye closure	N/R	N/R	N/R	−	Strabismus	N/R	Strabismus	−														
Facial dysmorphia	−	−	Ep fold + other	No eyebrows/laches	Hypertel, downslant palp fiss	Misc incl LSE, micrognathia	Ante. nostrils, long philtrum, LSE	Misc. incl dyspl ears, small pinched nose	N/R	Hypertel, retrognathia, LSE	Facial dysmorphisms	−	Ep fold/other	Retro‐gnathia	+	+														
Hearing impairment	−	−	N/R	N/R	−	N/R	N/R	N/R	N/R	N/R	N/R	N/R	N/R	−	N/R	N/R														
Internal organs																										
Hepatic	−	Micronodular cirrhosis, ↑ ALT, ↑AST	Hyperbilirubinemia, ↑ ALT,↑AST	Cholestasis	Hyperbilirubinemia, ↑ ALT,↑AST	↑ Liver enzymes	N/R	Hyperechogenic liver	N/R	Hyperbilirubinemia	Hyperbilirubinemia, ↑ ALT,↑AST	−	↑ ALT, ↑AST,↑γ‐GT	↑ ALT,↑AST	−	−														
Transferrin pattern	Type II	Type II	Type II	N/R	N/R	N/R	Type II	N/R	N/R	N/R	Type II	N/R	Type II	N/R	N/R	N/R														
Renal	−	−	−	Proximal tubulopathy	−	−	N/R	N/R	N/R	−	N/R	N/R	N/R	Kidney lesions	−	−														
Endocrinological	N/R	N/R	N/R	N/R	−	Cryptoorch, micropenis	Ambiguous genitalia	N/R	N/R	Ambiguous genitalia	Ambiguous genitalia	N/R	N/R	N/R	N/R	N/R														
Gastrointestinal	Normal ultrasound	−	Recto‐vaginal fistula, FTT, diarrhea	N/R	Chronic diarrhea	Umbilical + inguinal hernias	Vomiting, pseudoobstruction, FTT, TPN	N/R	N/R	Normal ultrasound, incomplete malrotation	Liver failure	Normal ultrasound	Normal ultrasound	Intestinal gas	N/R	N/R														
Cardiovascular																									
Cardiac	−	−	−	N/R	PFO/ASD	Pulmonary artery stenosis sin	−	Moderate cardiomegaly	N/R	−	Sub‐aortic VSD	VSD/PFO	−	VSD, ASD, PFO, tricuspid insuff	PDA/AV dysplasia	N/R														
Respiratory	−	−	N/R	N/R	−	Respiratory insufficiency	Chronic resp insuff; tracheostomy	Respiratory insuff, lung hypoplasia	Respiratory insufficiency	N/R	Respiratory insufficiency	−	−	Respiratory insufficiency	N/R	N/R														
Hema‐/immunology																								
Hematology/coagulation	−	↑ PT, trc ↓, splenomegaly	↓ Protein C	Pancytopenia, ‘coagulopathy’	−	‘Coagulopathy’ splenomegaly	N/R	trc ↓	N/R	N/R	↑ PT	↑ PT/D‐dimer	↑ PT/aPTT, trc ↓	↑ aPTT	N/R	N/R														
Immunology	Frequent infections	−	N/R	N/R	−	N/R	N/R	−	−	N/R	−	Recurrent fevers	HLH	Eosinophil dysf, resp insufficiency	N/R	N/R														
Connective tissue																							
Dermatological	Hyperkeratosis	Hyperkeratosis	N/R	Scaling and abrasions/erosions	Dry skin	N/R	N/R	Hyperkeratosis, dry skin, abrations, scaling	Dry, tight skin	N/R	Ichthyosis	N/A	−	Ectodermal dysplasia	Lipo‐dystrophy, IN	−														
Hypohidrosis	+	+	N/R	N/R	+	N/R	+	N/R	N/R	N/R	N/R	+	+	+	−	+														
Dental	Enamel hypoplasia	Enamel hypoplasia	N/R	Preeruption	N/R	N/R	N/R	Pre‐eruption	Pre‐eruption	Pre‐eruption	Pre‐eruption	−	Enamel hypoplasia	Molar hypocalcification	N/R	N/R														
Articular	Laxity	Laxity	Arthrogryposis	Arthrogryposis	Contractures	N/R	Distal arthrogryposis	Arthrogryposis	Arthrogryposis	Arthrogryposis	Arthrogryposis	N/R	N/R	N/R	Contractures	Contractures														
Skeletal	−	−	N/R	Arachno‐dactylyl, clubfoot	Thumbs adducted	Campto‐dactylyl, clubfeet	Bilateral clubfeet	Thoraco‐lumbal scoliosis	N/R	Kyphosis	Kyphoscoliosis	−	−	Scoliosis	Dysplasia	−														
	R11:3	R11:4	R11:5	R11:6	R11:7	R12:1	R13:1	R13:2	R13:3	R13:4	R13:5	R13:6	R13:7	R14:1	R15:1	R16:1														
Demography																						
Sex	F	M	F	F	M	N/R	F	M	F	M	M	F	F	M	F	F														
Origin	Saudi	Saudi	Saudi	Saudi	Saudi	N/R	Bulgarian	Turkish	Turkish	Moroccan	Moroccan	Moroccan	Turkish	Saudi	Moroccan	Turkish														
Deceased	Alive	Alive	Alive	Alive	Alive	8 days	26 days	12 months	15 months	Alive	14 months	5 weeks	Alive	Alive	Alive	5 weeks														
Genetics																					
Gene variant	hz. 11.67‐24A>G	hz. 1167‐24A>G	hz. 1167‐24A>G	hz. 1075‐9 T>G	hz. 1167‐24A>G	N/R	hz. 511C>T	hz. 1746+ 2 T > G	hz. 1238_1239 insA	1646G>T/ 785A>G	1646G>T/ 785A>G	N/R	511C>T/ 1746 + 2 T>G	hz. 1167‐24A > G	hz. 1646G>T	hz. 1646G>T														
Protein consequence	Gly390 Phefs*6	Gly390Phefs*6	Gly390Phefs*6	splice defect	Gly390Phefs*6	N/R	Arg171*	Splice defect	Phe414 Leufs*4	Gly549Val/ Tyr262Cys	Gly549Val/ Tyr262Cys	N/R	Arg171*/ splice defect	Gly390 Phefs*6	Gly549Val	Gly549Val														
GnomAD frequency	0	0	0	0	0	N/R	3.2E‐5	0	0/0	2.4E‐4/4E‐6	2.4E‐4/4E‐6	N/R	3.2E‐5/0	0	2.4E‐4	2.4E‐4														
Pregnancy/perinatal																				
Pregnancy	N/R	N/R	N/R	N/R	N/R	N/R	Poly‐hydramniosis	N/R	N/R	Oligo‐hydramniosis	N/R	N/R	N/R	−	−	−														
Gestational age at birth	Premature	Term	Premature	Term	Term	N/R	38	N/R	33	42	Term	41	37	Term	37	Term														
Peri−/neonatal event	N/R	N/R	N/R	N/R	N/R	N/R	Apgar 2–5‐5	N/R	−	C section	C section	N/R	N/R	RDS	−	−														
Growth																			
Head circumference	N/R	Microcephaly	Microcephaly	Microcephaly	Microcephaly	Microcephaly	Microcephaly	Microcephaly, <P3	Microcephaly, <P3	Microcephaly, <P3	Microcephaly	Microcephaly	Microcephaly, <P3	−	Microcephaly, −3.1 SD	N/R														
Length	N/R	N/R	N/R	N/R	N/R	N/R	N/R	P3‐P10	<P3	P3‐P10	−	N/R	N/R	−	−3.1SD	N/R														
Weight	N/R	N/R	N/R	N/R	N/R	Microsomia	Growth retardation	FTT, <P3	FTT, <P3	P3‐P10	−	N/R	growth retardation	−	FTT, −4.6 SD	N/R														
Neurological																		
Brain MRI	Reduced WM, thin corpus callosum, PVL	−	Delayed myelination, thin corpus callosum	Thin corpus callosum	Brain atrophy, PVL	N/R	Corpus callosum hypoplasia, hydrocephalus, abnormal gyration	Enlarged CSF spaces, asymmetrical lat ventricles	Cerebral and cerebellar atrophy	N/R	N/R	−	Cortical atrophy	N/R	N/R	Intracranial bleeding														
Developmental delay (DD)/ intellectual disability (ID)	DD	DD	DD	DD	DD	Indeterminable	Indeterminable	DD	DD	ID	DD	N/R	Severe ID	Mild ID, IQ 60	Mild ID	Indeterminable														
Seizures	−	−	−	−	−	N/R	Seizure	N/R	−	N/R	−	N/R	N/R	−	1 febrile seizure/EEG normal	Intractable seizures														
Muscular tone	Hypotonia	−	Hypotonia	Hypotonia	Hypotonia	Hypotonia	Hypotonia	Hypotonia	−	−	Hypotonia	Hypotonia	Hypotonia	−	Hypotonia	N/R														
Muscular	N/R	N/R	N/R	N/R	N/R	N/R	−	N/R	N/R	↑ CK	N/R	↑ CK	N/R	−	N/R	↑ CK														
Neuropsychiatric	N/R	N/R	N/R	Autism	N/R	N/R	N/R	N/R	N/R	N/R	N/R	N/R	Stereotypical/self‐harm	N/R	N/R	N/R														
Ophthalmological	−	−	−	−	Strabismus, bilat ptosis	N/R	Optic nerve atrophy	No response on ERG	Strabismus	N/R	N/R	N/R	N/R	−	−	N/R														
Facial dysmorphia	+	+	+	+	+	N/R	+	+	+	+	−	N/R	+	Mild	+	N/R														
Hearing impairment	N/R	N/R	N/R	N/R	N/R	N/R	N/R	Sensorineural loss	N/R	Conductive loss	N/R	−	−	−	N/R	N/R														
Internal organs																													
Hepatic	↑ ALT,↑AST	↑ ALT,↑AST	−	−	−	Fatal liver failure	Hepatomegaly, cholestasis	↑ ALT,↑AST	Hepatomegaly	↑ ALT,↑AST	Hepatomegaly, cholestasis, liver failure	Hepatomegaly, cholestasis	Hepatomegaly, cholestasis, cirrhosis	−	Micronodular cirrhosis, ↑ ALT,↑AST	↑ AST, cholestasis														
Transferrin pattern	N/R	N/R	N/R	N/R	N/R	Type II	Type II	Type II	Type II	Type II	Type II	N/R	Type II	−	Type II	Type II														
Renal	−	−	−	−	−	N/R	Hyper‐echogenic	−	N/R	−	N/R	−	Unilateral agenesis	−	Proximal tubulopathy	N/R														
Endocrinological	N/R	N/R	N/R	N/R	N/R	N/R	N/R	N/R	N/R	N/R	N/R	N/R	N/R	N/R	N/R	N/R														
Gastrointestinal	N/R	N/R	N/R	N/R	N/R	N/R	N/R	Chronic diarrhea	Chronic diarrhea	N/R	−	Bowel ischemia	Chronic diarrhea	−	IBD, normal ultrasound	Severe vomiting														
Cardiovascular																	
Cardiac	N/R; **ALT, alanine aminotransferase; aPTT, activated partial thromboplastin time; ASD, atrial septal defect; AST, aspartate aminotransferase; AV, aortic valve; BRP, birth‐related problems; CK, creatine kinase; DD	N/R	N/R	N/R	N/R	N/R	ASD/PDA	N/R	ASD/PDA	N/R	N/R	ASD	VSD	−	N/R	N/R														
Respiratory	N/R	N/R	N/R	N/R	N/R	N/R	N/R	N/R	N/R	N/R	N/R	N/R	N/R	N/R	N/R	N/R														
Hema‐/immunology															
Hematology/coagulation	N/R	N/R	N/R	N/R	N/R	‘Coagulopathy’	Splenomegaly	Trc ↓	Splenomegaly, trc ↓	Splenomegaly,aPTT↑, FXI↓, trc↓	Splenomegaly, aPTT ↑, trc ↓	Splenomegaly	Splenomegaly, pancytopenia, aPTT ↑ PT ↑	−	−	Vitamin K deficiency														
Immunology	−	−	−	−	−	−	−	Frequent infections	Frequent infections	Frequent infections	Frequent infections	−	Frequent infections	Frequent infections	Frequent infections	−														
Connective tissue																
Dermatological	−	−	−	Lipodystrophy	−	N/R	N/R	N/R	Hyper‐keratosis	Hyperkeratosis	Dry skin	N/R	Orange peel skin	Hyper‐keratosis	N/R	N/R														
Hypohidrosis	+	+	−	−	+	Hyperthermia	Hyperthermia	N/R	N/R	+	+	+	+	+	N/R	N/R														
Dental	N/R	N/R	N/R	N/R	N/R	Pre‐eruption	Pre‐eruption	N/R	N/R	Missing teeth	N/R	Pre‐eruption	Caries	Enamel hypoplasia	N/R	Pre‐eruption														
Articular	Contractures	Contractures	Contractures	Contractures	Contractures	Arthrogryposis	Arthrogryposis	N/R	N/R	N/R	N/R	N/R	Hypermobility	−	N/R	N/R														
Skeletal	−	Dysplasia	−	−	−	N/R	N/R	N/R	Postaxial polydactylyl	−	−	−	Scoliosis	−	Postaxial polydactylyl	N/R														

*Note*: Features of the two COG6‐CDG patients in our cohort (P1 and P2) and the previously published cases (R1:1, Cirnigliaro et al.^6^; R2:1, Ververi et al.^7^; R3:1, Zhao et al.^8^; R4:1, Lugli et al.^10^; R5:1, Lugli et al.^9^; R6:1, R6:2, Komlosi et al.^11^; R7:1, R7:2, Mandel et al.^12^; R8:1, Li et al.^13^; R9:1, Althonaian et al.^14^; R10:1, Wu et al.^15^; R11:1‐R11:7, Alsubhi et al.^16^; R12:1; Pérez‐Cerdá et al.^17^; R13:1‐R13:7, Rymen et al.^18^; R14:1, Shaheen et al.^19^; R15:1, Huybrechts et al.^20^; R16:1, Lubbehusen et al.^21^ In Shaheen et al.,^19^ 11 more patients are mentioned but not individually clinically described).

Abbreviations: ALT, alanine aminotransferase; aPTT, activated partial thromboplastin time; ASD, atrial septal defect; AST, aspartate aminotransferase; AV, aortic valve; BRP, birth‐related problems; CK, creatine kinase; DD, developmental delay; ep, epicanthal; F, female; FTT, failure to thrive; FXI, factor 11; HLH, hemophagocytic lymphohistiocytosis; ID, intellectual disability; IN, inverted nipples; LSE, low‐set ears; M, male; P, percentile; PDA, persistent ductus arteriosus; PFO, persistent foramen ovale; PT, prothrombin time; PVL, periventricular leukomalacia; SD, standard deviation; trc, thrombocytes; US, ultrasound; VSD, ventricular septal defect; WM, white matter; ‐, not present; +, present, N/R, not reported.

COG6‐CDG entails two different phenotypes: one very severe including intrauterine growth retardation and polyhydramniosis, arthrogryposis, and contractures, often premature delivery, severe developmental and growth issues, liver pathology and early death; and one milder with normal pregnancies, mild to moderate developmental delay, transaminitis, chronic diarrhea, enamel hypoplasia, hypohidrosis with heat sensitivity, hyperkeratosis, and neuropsychiatric disorders. Approximately 45% of the compound heterozygous patients compared to 34% of the homozygous ones were reported deceased at the time of the report. However, most surviving homozygous patients (16/21) were from the same two families,^16^, ^19^ carrying the same variant (c.1167‐24A>G), thus skewing the data.

Neurological disease is an important finding in most CDG,^3^ including COG6‐CDG. A substantial number of the patients had an MRI reported (26/43) of which 21 showed at least one pathological feature. The most common was agenesis or hypoplasia of the corpus callosum (10/21), and brain atrophy causing enlarged ventricles (8/21). On the other hand, in other CDG,^3^ cerebellar atrophy is a common feature, but in COG6‐CDG it was only seen in 4/21. P1 showed delayed myelinization whereas P2 had a normal MRI scan, which is uncommon (3/25 in the patient cohort). Microcephaly is a prominent feature of COG6‐CDG found in 22/25 with reported head growth measurements. Furthermore, epilepsy is common in CDG,^3^ and in many types, this is a main feature,^26^ causing a significant disease burden. However, in COG6‐CDG it seems a lesser problem as only 5/29 were reported to have or have had epilepsy^9^, ^13^, ^16^, ^18^, ^21^ and only one was pharmacoresistant. Developmental delay/cognitive impairment is a hallmark of COG6‐CDG and no patients with normal cognition have been reported, the intellectual disability ranges from mild to severe in tested patients. We carefully characterized the cognitive and neuropsychiatric function of the patients and describe ADHD‐like features in P1, not previously described. Other non‐neurological features of COG6‐CDG reported previously include hypohidrosis and heat sensitivity, hyperkeratotic skin disorders, enamel hypoplasia, and a spectrum from full‐blown arthrogryposis multiplex congenital to joint laxity.^18^ The patients described here also presented these features, which further strengthens the notice that these findings seem to be hallmark signs of COG6‐CDG and that their presence in a child with neurological symptoms should prompt testing for deficient glycosylation.

Neurologically, the novel patients described in this article are in the mild spectrum of the disease, but despite this, there are clear biochemical findings of a delayed both antero‐ and retrograde transport within the ER‐Golgi vesicular system, proving that this can be used to validate pathogenicity of mutations previously deemed uncertain. This assay has previously been shown to ascertain pathogenicity in COG7‐CDG.^27^ It has been reported that COG6 depletion can cause instability of the other lobe B COG subunits in both COG6‐CDG patients and other cancer cell lines.^21^, ^28^, ^29^ For example, in individuals homozygous for the pathogenic variant c.1646G>T (p. Gly549Val), there is an 80% reduction of COG6 protein, causing decreased COG5 and COG7 protein levels.^21^ In HEK293T and HELA cells, completely knocking out COG6 protein causes less steady‐state level of COG5, COG7, and COG8.^28^, ^29^ We observed a similar effect of reduced COG6 protein on COG7 in our two patients, with about approximately 50% depletion of COG7 protein level. We also observed a significant decrease in COG8 protein in P2, which shows more than 90% depletion of COG6. A deficiency in COG6 is known to affect multiple glycosylation pathways,^20^ however, we only performed analysis of the *N*‐glycosylation status of transferrin, which showed a typical type 2 pattern. It is noteworthy though that a normal transferrin glycosylation test does not preclude this disorder.^19^

In conclusion, we describe in detail two patients with COG6‐CDG, one of which is the first patient of Nordic/South American descent. Reviewing data on 41 published patients confirms the presence of two clinically different groups, one milder and one very severe including early death. We show that a combination of Western blotting of the different COG components in conjunction with an assay of retro‐ and anterograde ER‐Golgi transport is useful to confirm the pathogenicity of mutations in the COG6 gene, of particular interest in milder cases with variants of uncertain significance.

## AUTHOR CONTRIBUTIONS

Zhi‐Jie Xia analyzed and interpreted data, and revised the manuscript; Bobby G. Ng took part in design, analysis and interpretation of data, and revised the manuscript; Elizabeth Jennions, had patient responsibility, analyzed and interpreted data, and revised the manuscript; Maria Blomqvist analyzed and interpreted data, and revised the manuscript; Anneli Sandqvist Wiklund analyzed and interpreted data, and revised the manuscript; Carola Hedberg‐Oldfors analyzed and interpreted data, and revised the manuscript; Carlos Rodriguez Gonzalez analyzed and interpreted data, and revised the manuscript; Hudson H. Freeze took part in project design and revised the manuscript; Sofia Ygberg took part in design, had patient responsibility, interpreted data and revised the manuscript; Erik A. Eklund conceptualized and designed the project, and drafted the manuscript.

## FUNDING INFORMATION

The study was funded from The Rocket Fund and R01DK99551 to HHF and SUS Stiftelser och donationer to EAE. The authors confirm independence from the sponsors; the content of the article has not been influenced by the sponsors.

## CONFLICT OF INTEREST

The authors declare no conflict of interest.

## ETHICS STATEMENT

All procedures followed were in accordance with the Helsinki Declaration of 1975, as revised in 2000.

## INFORMED CONSENT

Informed consent was obtained from all individuals being included in the study (Sanford Burnham Prebys Medical Discovery Institute [IRB‐2014‐038‐17]).

## Data Availability

Original data can be made available upon request to the corresponding author.
